# Network Modeling of Liver Metabolism to Predict Plasma Metabolite Changes During Short-Term Fasting in the Laboratory Rat

**DOI:** 10.3389/fphys.2019.00161

**Published:** 2019-03-01

**Authors:** Kalyan C. Vinnakota, Venkat R. Pannala, Martha L. Wall, Mohsin Rahim, Shanea K. Estes, Irina Trenary, Tracy P. O’Brien, Richard L. Printz, Jaques Reifman, Masakazu Shiota, Jamey D. Young, Anders Wallqvist

**Affiliations:** ^1^Henry M. Jackson Foundation for the Advancement of Military Medicine, Inc., Bethesda, MD, United States; ^2^Department of Defense Biotechnology High Performance Computing Software Applications Institute, Telemedicine and Advanced Technology Research Center, United States Army Medical Research and Materiel Command, Fort Detrick, MD, United States; ^3^Department of Chemical and Biomolecular Engineering, Vanderbilt University School of Engineering, Nashville, TN, United States; ^4^Department of Molecular Physiology and Biophysics, Vanderbilt University School of Medicine, Nashville, TN, United States

**Keywords:** metabolic network, rat, liver, plasma, metabolomics, fasting, central carbon flux, gluconeogenesis

## Abstract

The liver—a central metabolic organ that integrates whole-body metabolism to maintain glucose and fatty-acid regulation, and detoxify ammonia—is susceptible to injuries induced by drugs and toxic substances. Although plasma metabolite profiles are increasingly investigated for their potential to detect liver injury earlier than current clinical markers, their utility may be compromised because such profiles are affected by the nutritional state and the physiological state of the animal, and by contributions from extrahepatic sources. To tease apart the contributions of liver and non-liver sources to alterations in plasma metabolite profiles, here we sought to computationally isolate the plasma metabolite changes originating in the liver during short-term fasting. We used a constraint-based metabolic modeling approach to integrate central carbon fluxes measured in our study, and physiological flux boundary conditions gathered from the literature, into a genome-scale model of rat liver metabolism. We then measured plasma metabolite profiles in rats fasted for 5–7 or 10–13 h to test our model predictions. Our computational model accounted for two-thirds of the observed directions of change (an increase or decrease) in plasma metabolites, indicating their origin in the liver. Specifically, our work suggests that changes in plasma lipid metabolites, which are reliably predicted by our liver metabolism model, are key features of short-term fasting. Our approach provides a mechanistic model for identifying plasma metabolite changes originating in the liver.

## Introduction

The liver is the primary organ responsible for metabolizing drugs and toxicants, a process collectively known as xenobiotic metabolism. This function makes the liver highly susceptible to injury and potential failure (Zimmerman, 1999). Current clinical markers of liver cell damage, such as the enzymes alanine amino transferase (ALT) and aspartate amino transferase (AST), which appear one to several days following exposure to a toxicant, are often limited in sensitivity and specificity to detect the pathology or injury (Zimmerman, 1999). Metabolite profiles, as measured in the plasma and urine of laboratory animal models of liver injury, are actively being investigated for their potential to detect liver damage earlier than current clinical markers and thereby facilitate timely intervention (Kamp et al., 2012; Mattes et al., 2014; Beger et al., 2015; Iruzubieta et al., 2015; Chang et al., 2017; Jarak et al., 2017). Additionally, they are being analyzed to identify canonical metabolic pathways (i.e., not including xenobiotic metabolism), such as lipid, amino acid, and oxidative stress pathways, which are perturbed during a drug- or toxicant-induced liver injury. However, plasma metabolite profiles and canonical metabolic pathways are also affected by the nutritional and physiological state of an animal, which could confound the identification of liver injury-induced changes in the plasma metabolite profile (Mellert et al., 2011; Mu et al., 2015). Importantly, the plasma metabolite profile consists of contributions from all other organs in the body, each of which is determined by the physiological state of the organ. It is important, therefore, to identify the contributions of liver metabolism to the plasma metabolite profile, and the metabolic pathways contributing to the observed changes under physiological and pathophysiological perturbations.

Genome-scale computational modeling of organ metabolism constitutes an important approach toward obtaining mechanistic insights into organ metabolism and canonical metabolic pathways under various conditions (Blais et al., 2017). Here, we subjected rats to short-term fasting in vehicle control groups of a larger study involving three different toxicants, and applied a genome-scale rat metabolic network to assess liver contributions to plasma metabolite profiles and to identify the responsible metabolic pathways. The short-term fasting conditions studied here were dominated by hormonally regulated changes in liver glycogen breakdown without significant transcriptomic changes of liver enzymes, which created a challenge in applying a genome-scale network modeling approach to describe liver function. We made our modeling analysis represent the liver mainly by constraining the model with the measured metabolic fluxes in this study and fluxes reported in the literature under similar conditions. Specifically, we measured the evolution of key metabolic fluxes in the liver, the liver transcriptome, and plasma metabolite profiles in three *in vivo* studies during which the rats underwent short-term food deprivation for up to 13 h. We used a recently published algorithm to integrate the measurements with a rat metabolic network model, and predicted the direction of change in extracellular metabolite concentrations resulting from a perturbation of metabolic fluxes in the network (Blais et al., 2017; Pannala et al., 2018). By comparing model predictions of the directions of metabolite changes with measured plasma metabolite profiles, we assessed the contributions of the liver to those changes.

## Materials and Methods

### Animals and Study Groups

Male Sprague-Dawley rats at 10 weeks of age were purchased from Charles River Laboratories (Wilmington, MA, United States). The rats were fed with Formulab Diet 5001 (Purina LabDiet; Purina Miles, Richmond, IN, United States) and given water *ad libitum* in an environmentally controlled room with a 12:12-h light-dark cycle at 23°C. All experiments were conducted in accordance with the Guide for the Care and Use of laboratory Animals of the United States Department of Agriculture, using protocols approved by the Vanderbilt University Institutional Animal Care and Use Committee, and by the United States Army Medical Research and Materiel Command Animal Care and Use Review Office.

Three types of measurements, plasma metabolite profiles, liver gene expression, and stable isotope tracer-based metabolic flux profiles, were made at one or two time points in three experimental studies. The three studies described here were the vehicle control groups of a larger study involving three different toxicants. The vehicle for each toxicant was different due to their differing physical and chemical properties. The time points also varied slightly because of the differences in their toxicity in the larger study. Table 1 summarizes the number of animals for each measurement in each study.

**Table 1:** Number of animals used for each measurement per time point in Studies 1–3.

Measurement	Study 1		Study 2		Study 3	
			
	5 h	10 h	5 h	10 h	7 h	13 h
Metabolic flux	–	9	–	8	–	8
Plasma metabolite profiles	8	8	8	8	9	9
Liver gene expression	8	8	8	8	8	8


### Catheter Implantation for Infusions and Sampling

Catheter implantation surgery was performed 7 days before each experiment, as previously described (Shiota, 2012). Rats were anesthetized with isoflurane, after which one of two procedures was performed depending on the type of measurement to be collected during the experiment. To measure changes in gene expression and plasma metabolite profiles, the right external jugular vein was cannulated with a sterile silicone catheter [0.51 mm inner diameter (ID) and 0.94 mm outer diameter (OD)]. Alternatively, to measure metabolic flux, both the carotid artery and the right external jugular vein were cannulated with sterile silicone catheters (0.51 mm ID and 0.94 mm OD). The free ends of the implanted catheters were passed subcutaneously to the back of the neck, where they were fixed. Finally, each implanted catheter was occluded with a metal plug after a flush with heparinized saline solution (200 U heparin/ml). The rats were housed individually after the surgery.

### Procedures for Measuring Changes in Gene Expression and Plasma Metabolite Profiles

Two time points were selected for sampling tissue and blood after vehicle administration in each of the three studies analyzed in the present paper: they were 5 h and 10 h for Studies 1 and 2, and 7 h and 13 h for Study 3. The administered vehicles and their dosages were polyethylene glycol at 6 ml/kg, corn oil at 2 ml/kg, and saline at 2 ml/kg in Studies 1, 2, and 3, respectively. Following blood collection, animals were given vehicle by oral gavage at 7 a.m. and moved to a new housing cage, where they were given access to water *ad libitum* but not food. Then, at 12 p.m. (5 h group) or 5 p.m. (10 h group), after blood collection, animals in Studies 1 and 2 were anesthetized by intravenous injection of sodium pentobarbital through the jugular vein catheter and immediately subjected to laparotomy. The same procedures were performed at 2 p.m. (7 h group) or 8 p.m. (13 h group) in Study 3. After laparotomy, the liver was dissected and frozen using Wollenberger tongs precooled in liquid nitrogen. The collected plasma and liver samples were stored at -80°C until use for further analyses.

### Methods for Measuring Metabolite Flux

#### *In vivo* Procedures in the Rat

At 7 a.m. on the day of the study, rats in all three studies were administered vehicle (50% polyethylene glycol or 6 ml/kg of either saline or corn oil) by oral gavage. Then, after food and water were removed, they were anesthetized with isoflurane at 12:50 p.m. (Studies 1 and 2) or 3:50 p.m. (Study 3). Subsequently, a 200-μl arterial blood sample was collected through the carotid artery catheter to determine the natural isotopic abundance of circulating glucose, after which a bolus of [^2^H_2_]water (99.9%) was delivered subcutaneously to enrich total body water to 4.5%. A [6,6-^2^H_2_]glucose prime (80 mg ⋅ kg^-1^) was dissolved in the bolus. Post-awakening, at 1 p.m. or 4 p.m. (i.e., 6 or 9 h after dosing), rats were connected to sampling and infusion lines and placed in bedded containers without food or water. Following the bolus, [6,6-^2^H_2_]glucose was administered as a continuous infusion (0.8 mg ⋅ kg^-1^ ⋅ min^-1^) into the systemic circulation through the jugular vein catheter for the duration of the study. Sodium [^13^C_3_]propionate (99%) was delivered as a primed (110 mg ⋅ kg^-1^), continuous (5.5 mg ⋅ kg^-1^ ⋅ min^-1^) infusion starting 120 min after delivery of the [^2^H_2_] water bolus. All infusates were prepared in a 4.5% [^2^H_2_] water-saline solution unless otherwise specified. Stable isotopes were obtained from Cambridge Isotope Laboratories (Tewksbury, MA, United States). Blood glucose was monitored (AccuCheck; Roche Diagnostics, Indianapolis, IN, United States) and donor erythrocytes were infused to maintain hematocrit throughout the study. Three blood samples (300 μl each) were collected over a 20-min period following 100 min of [^13^C_3_]propionate infusion. Arterial blood samples were centrifuged in EDTA-coated tubes for plasma isolation, and the three 100-μl plasma samples were stored at -20°C prior to glucose derivatization and gas chromatography-mass spectrometry (GC-MS) analysis. Rats were rapidly euthanized through the carotid artery catheter immediately after the final steady-state sample was collected.

#### Preparation of Glucose Derivatives

Plasma samples were divided into three aliquots and derivatized separately to obtain di-*O*-isopropylidene propionate, aldonitrile pentapropionate, and methyloxime pentapropionate derivatives of glucose. For di-*O*-isopropylidene propionate preparation, proteins were precipitated from 20 μl of plasma using 300 μl of cold acetone, and the protein-free supernatant was evaporated to dryness in screw-cap culture tubes. Derivatization proceeded as described previously (Antoniewicz et al., 2011) to produce glucose 1,2,5,6-di-isopropylidene propionate. For aldonitrile and methyloxime derivatization, proteins were precipitated from 10 μl of plasma using 300 μl of cold acetone and the protein-free supernatants were evaporated to dryness in microcentrifuge tubes. Derivatizations then proceeded as described previously (Antoniewicz et al., 2011) to produce glucose aldonitrile pentapropionate and glucose methyloxime pentapropionate. All derivatives were evaporated to dryness, dissolved in 100 μl of ethyl acetate, and transferred to GC injection vials with 250 μl glass inserts for GC-MS analysis.

#### GC-MS Analysis

GC-MS analysis was performed using an Agilent 7890A gas chromatography system with an HP-5 ms (30 m × 0.25 mm × 0.25 μm, Agilent J&W Scientific; Agilent Technologies Inc., Santa Clara, CA, United States) capillary column interfaced with an Agilent 5975C mass spectrometer. Samples were injected into a 270°C injection port in splitless mode. Helium flow was maintained at 0.88 ml ⋅ min^-1^. For analysis of di-*O*-isopropylidene and aldonitrile derivatives, the column temperature was held at 80°C for 1 min, ramped at 20°C ⋅ min^-1^ to 280°C and held for 4 min, then ramped at 40°C ⋅ min^-1^ to 325°C. For methyloxime derivatives, the same oven program was used except the ramp to 280°C was 10°C ⋅ min^-1^. After a 5 min solvent delay, the MS collected data in scan mode from m/z 300 to 320 for di-*O*-isopropylidene derivatives, m/z 100 to 500 for aldonitrile derivatives, and m/z 144 to 260 for methyloxime derivatives. Each derivative peak was integrated using a custom MATLAB^®^(Mathworks Inc., Natick, MA, United States) function (Antoniewicz et al., 2007) to obtain mass isotopomer distributions (MIDs) for six specific ion ranges: aldonitrile – m/z 173–177, 259–265, 284–288, 370–374; methyloxime – m/z 145–149; di-*O*-isopropylidene – m/z 301–308. To assess uncertainty, root mean square error was calculated by comparing the baseline MID of unlabeled glucose samples to the theoretical MID computed from the known abundances of naturally occurring isotopes.

#### ^2^H/^13^C Metabolic Flux Analysis (MFA)

A detailed description of the *in vivo* metabolic flux analysis methodology employed in these studies has been previously provided (Hasenour et al., 2015). Briefly, a reaction network was constructed using the INCA software package (Young, 2014). The reaction network defined the carbon and hydrogen transitions for biochemical reactions linking hepatic glucose production and associated intermediary metabolism reactions. Flux through each reaction was estimated relative to citrate synthase (fixed at 100) by minimizing the sum of squared residuals between simulated and experimentally determined MIDs of the six fragment ions previously described. Flux estimation was repeated at least 25 times from random initial values. Goodness-of-fit was assessed by a chi-square test, and 95% confidence intervals were computed by evaluating the sensitivity of the sum-of-squared residuals to variations in flux values (Antoniewicz et al., 2006). The average sum of squares of residuals (SSR) of each experimental group fell within the 95% confidence interval of the corresponding chi-square distribution with *D* degrees of freedom: Study 1 (*D* = 22): SSR = 29.65 ± 7.05; Study 2 (*D* = 23): SSR = 28.77 ± 2.83; Study 3 (*D* = 26): SSR = 22.69 ± 1.83. Relative fluxes were converted to absolute values using the known [6,6-^2^H_2_]glucose infusion rate and rat weights. Flux estimates for the steady-state samples were averaged to obtain a representative set of values for each rat.

### Metabolomic Analysis

#### Sample Preparation and Ultrahigh Performance Liquid Chromatography/Mass Spectrometry (UHPLC/MS)

Sample preparation was carried out at Metabolon, Inc. in a manner similar to a previous study (Hatano et al., 2016). Briefly, individual samples were subjected to methanol extraction and then split into aliquots for analysis by UHPLC/MS. The global biochemical profiling analysis comprised four unique arms: reverse phase chromatography positive ionization methods optimized for hydrophilic compounds (LC/MS Pos Polar) and hydrophobic compounds (LC/MS Pos Lipid); reverse phase chromatography with negative ionization conditions (LC/MS Neg), and a hydrophilic interaction liquid chromatography (HILIC) method coupled to negative ionization (LC/MS Polar) (Evans et al., 2014). All of the methods alternated between full scan MS and data-dependent MS*^n^* scans. The scan range varied slightly between methods but generally covered 70–1,000 m/z.

Metabolites were identified by automated comparison of the ion features in the experimental samples to a reference library of chemical standard entries that included retention time, molecular weight (m/z), preferred adducts, and in-source fragments as well as associated MS spectra, and curated by visual inspection for quality control using software developed at Metabolon. Identification of known chemical entities was based on comparison to metabolomic library entries of purified standards (Dehaven et al., 2010).

#### Statistical Analysis of Metabolomic Data

We performed statistical analysis to identify metabolites that changed significantly with the duration of fasting. The raw data consisted of MS counts for each metabolite detected in a given plasma sample. We imputed any missing values with the minimum observed value for each metabolite. We then computed distributions of fold-change values for each metabolite and pooled them across the three studies to resolve changes during short-term fasting above experimental and biological noise. From these pooled distributions, we calculated 99% confidence intervals for the mean fold-change values of each metabolite using the percentile approach (Efron and Hastie, 2016). Briefly, for each metabolite at each of the two points in a study, we constructed *n* × 10^5^ instances of MS count data by random sampling with replacement, where *n* is the number of animals. Then, for each metabolite in the given study, we calculated *n* × 10^5^ fold-change values from the synthetic data sets generated in the previous step. We pooled these fold-change values across studies for a given metabolite, and calculated 10^5^ sample means, which constitute the bootstrapped distribution of the mean fold-change. To obtain the 99% confidence interval of the mean fold value for each metabolite, we identified a percentile-based confidence interval from the bootstrapped distribution of the mean fold-change value, which excluded values above the highest 0.5th percentile and those below the lowest 0.5th percentile. A metabolite was determined to have significantly increased or decreased if both bounds of the 99% confidence interval of its mean fold-change value were above or below the value 1. All statistical analyses were performed in MATLAB^®^R2017b (Mathworks Inc., Natick, MA, United States). We have provided MATLAB code for this analysis in the Supplementary Material.

### RNA Sequencing and Data Analysis

#### RNA Isolation and Sequencing

Total RNA was isolated from the liver, using TRIzol Reagent (Thermo Fisher Scientific, Waltham, MA, United States) and the direct-zol RNA Mini Prep kit (Zymo Research, Irvine, CA, United States). The isolated RNA samples were then submitted to the Vanderbilt University Medical Center VANTAGE Core (Nashville, TN, United States) for RNA quality determination and sequencing. Total RNA quality was assessed using a 2100 Bioanalyzer (Agilent, Santa Clara, CA, United States). At least 200 ng of DNase-treated total RNA with high RNA integrity was used to generate poly-A-enriched mRNA libraries, using KAPA Stranded mRNA sample kits with indexed adaptors (Roche, Indianapolis, IN, United States). Library quality was assessed using the 2100 Bioanalyzer (Agilent), and libraries were quantitated using KAPA library Quantification kits (Roche). In Study 1, pooled libraries were subjected to 75-bp single-end sequencing according to the manufacturer’s protocol (Illumina HiSeq 3000, San Diego, CA, United States). In contrast, in Studies 2 and 3, the respective pooled libraries were subjected to 150-bp paired-end sequencing on Illumina NovaSeq 6000 and 75-bp paired-end sequencing on Illumina HiSeq 3000 according to the manufacturer’s protocol. Bcl2fastq2 Conversion Software (Illumina) was used to generate de-multiplexed Fastq files.

#### Analysis of RNA-Seq Data

Analysis of RNA-seq data consists of two stages: (1) determination of transcript abundance and (2) determination of differentially expressed genes. We determined transcript abundance from Fastq files, consisting of raw sequence reads, using a recently published software tool Kallisto (Bray et al., 2016). Using Kallisto, we first generated a reference transcriptome index from cDNA files based on genome assembly Rnor6.0 for rat, published on ENSEMBL Release 92 (Zerbino et al., 2018). We then determined transcript abundance using Kallisto, which is based on pseudoalignment of raw sequence reads to the reference transcriptome index. We used appropriate Kallisto settings for processing single-end sequence reads from Study 1, and paired-end sequence reads from Studies 2 and 3. Using these transcription data, expressed in units of transcripts per million (TPM), we used the analytical tool Sleuth (Pimentel et al., 2017) to investigate differential expression of genes between two time points in each. Within Sleuth, we applied a likelihood ratio test to identify statistically significant gene expression changes and a Wald test to compute the effect sizes (logarithms of the fold-changes), between the two time points in each study, for each test. From these results, we obtained effect sizes for the genes that were identified by the likelihood ratio test to have changed significantly. Finally, we designated the genes with absolute effect sizes in the top 10th percentile as biologically significant, conditional upon statistical significance.

### Curation of Rat Metabolic Network *iRno* and Assignment of Physiological Flux Bounds

We first updated a recently published functional rat genome-scale network reconstruction *iRno*, which contains 2,325 genes and 5,620 metabolites in 8,336 reactions and eight compartments connected by Gene-Protein-Reaction rules, and is capable of simulating 327 liver-specific metabolic functions (Blais et al., 2017). The updates to *iRno* included additional reactions or modification of existing reactions based on experimental evidence (Supplementary Table S1). For instance, we removed a reaction (*S*)-lactate:ferricytochrome-c 2-oxidoreductase, which was determined to be non-existent in mammalian systems. Additionally, we added 90 transport and 105 exchange reactions to *iRno* to improve its coverage of exchangeable metabolites that were detected in plasma metabolite profiles in the present study. The updated *iRno* contains 2,325 genes and 5,709 metabolites including 3,201 unique metabolites in 8,534 reactions including 595 exchange reactions in eight compartments. Supplementary Table S1 provides the updated *iRno*.

The liver operates in a gluconeogenic mode during the short-term fasting trajectory in the present study. In this state, the liver takes up amino acids, lactate, and glycerol to produce glucose and urea. The liver also takes up non-esterified fatty acids to produce ketone bodies. We constrained the uptake rates of amino acids, fatty acids, lactate, and glycerol, using values reported in the literature from *in vivo* measurements in rats undergoing short-term fasting (Supplementary Table S2).

### Application of Transcriptionally Inferred Metabolic Biomarker Response (TIMBR) Algorithm

Transcriptionally inferred metabolic biomarker response (TIMBR) is a recently published method developed for predicting changes in extracellular metabolites due to gene expression changes under defined physiological operating conditions by integrating those changes into genome-scale network reconstructions (see Blais et al., 2017 for details). In the present study, we applied TIMBR to predict metabolite changes during a 5–6-h window of short-term fasting, where gene expression changes have little influence on metabolic state (Ikeda et al., 2014), in contrast to the changes in the central carbon metabolism fluxes. TIMBR calculates the global network demand required for producing a metabolite (*X*_met_) by minimizing the weighted sum of fluxes across all reactions for each condition and metabolite, while satisfying the steady-state mass balance and a defined optimal fraction of maximum network production flux capability (*ν*_opt_) to produce a metabolite as shown below:

(1) $$\begin{array}{l} X_{\mathrm{met}}=\min\sum \left|v\right|\\ s.t.\;:\;vx\geq v_{\mathrm{opt}};\;v_{\mathrm{lb}}\;<\;v\;<\;v_{\mathrm{ub}};\;S\cdot v=0 \end{array}$$

where ν is a vector of reaction fluxes and *S* is the stoichiometric matrix. We included boundary conditions for uptake and secretion rates into the algorithm by fixing the respective lower (*ν*_lb_) and upper bounds (*ν*_ub_) of the metabolite exchange reactions (*ν*_ex_), as shown in Eq. (2). Similarly, we integrated measurements from ^13^C-labeled tracer studies for some of the central carbon metabolism fluxes into the TIMBR algorithm by constraining the lower and upper bounds of the respective reactions in the model (*ν*_mfa_) (Eq. 3).

(2) $$v_{\mathrm{lb}}\;<\;v_{\mathrm{ex}}\;<\;v_{\mathrm{ub}}$$

(3) $$v_{\mathrm{lb}}\;<\;v_{\mathrm{mfa}}\;<\;v_{\mathrm{ub}}$$

Using this method, we determined the relative production scores for all metabolites (*X*_raw_) from 5 to 7 h (*X*_5-7_) and 10 to 13 h (*X*_10-13_) time points (Eq. 4), and then calculated the TIMBR production scores (*X*_s_) as the z-transformed scores across all exchangeable metabolites (Eq. 5).

(4) $$X_{\mathrm{raw}}=\frac{X_{5-7}-X_{10-13}}{X_{5-7}+X_{10-13}}$$

(5) $$X_{s}=\frac{X_{\mathrm{raw}}-\mu }{\sigma }$$

**FIGURE 1:**
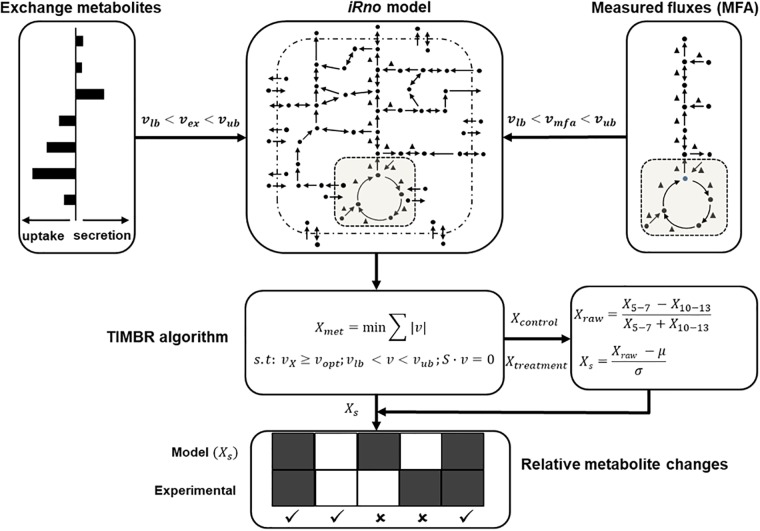
Schematic (adapted from Pannala et al., 2018) illustrates how we integrated physiological flux bounds for exchangeable metabolites (*ν*_ex_), measured central carbon fluxes (*ν*_mfa_) with the rat metabolic network model *iRno* to compute global network demand (*X*_met_) using TIMBR, by minimizing the sum of the absolute value of flux across all reactions at the earlier (*X*_5-7_) and later (*X*_10-13_) time points. We then calculated a z-transformed TIMBR score (*X*_s_) from the raw metabolite production score (*X*_raw_) for each metabolite, whose positive or negative sign indicated its predicted tendency to increase or decrease in plasma. The TIMBR scores were compared with the measured fold-change values of significantly changed metabolites in the plasma to assess the contributions of liver metabolism to those changes.

Figure 1 shows the workflow for the application of the TIMBR algorithm (adapted from Pannala et al., 2018). We performed the model computations in MATLAB R2017b using the linear programming solver provided in the GNU Linear Programming Kit. We refer the reader to the original publication for detailed descriptions of the TIMBR algorithm and the corresponding computer codes (Blais et al., 2017).

## Results and Discussion

### Liver Glucose Production and Glycogenolysis Fluxes Decrease With Fasting Duration

**FIGURE 2:**
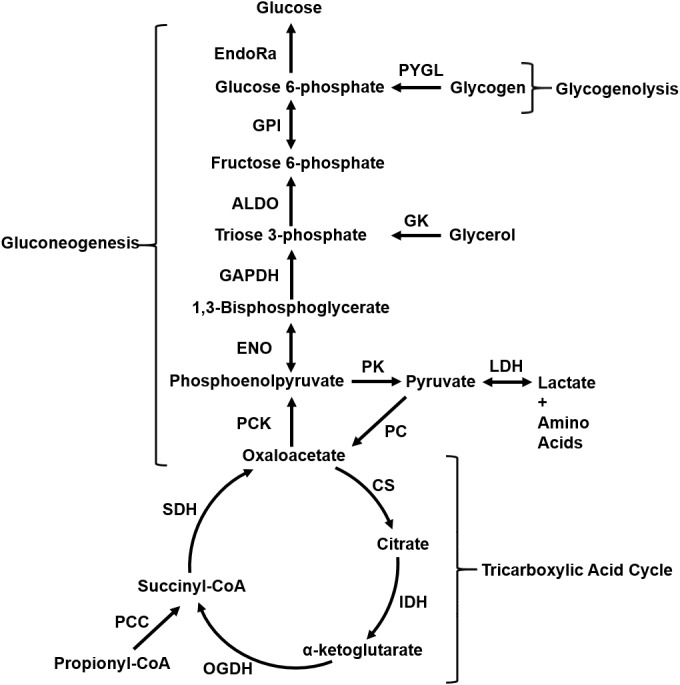
Figure (adapted from Pannala et al., 2018) depicts liver central carbon metabolism pathways that produce glucose by breaking down glycogen (glycogenolysis) and by gluconeogenesis from glycerol, lactate, and amino acids during fasting. Unidirectional arrows indicate reactions that operate far from thermodynamic equilibrium and are practically irreversible. Bidirectional arrows indicate reactions that operate closer to thermodynamic equilibrium and are reversible under physiological conditions. ALDO, aldolase; CS, citrate synthase; EndoRa, endogenous liver glucose production; ENO, enolase; GAPDH, glyceraldehyde phosphate dehydrogenase; GK, glycerol kinase; GPI, glucose-6-phosphate isomerase; IDH, isocitrate dehydrogenase; LDH, lactate dehydrogenase; OGDH, oxoglutarate dehydrogenase; PC, pyruvate carboxylase; PCC, propionyl-CoA carboxylase; PCK, phos-phosphoenolpyruvate carboxykinase; PK, pyruvate kinase; PYGL, glycogen phosphorylase; SDH, succinate dehydrogenase.

**FIGURE 3:**
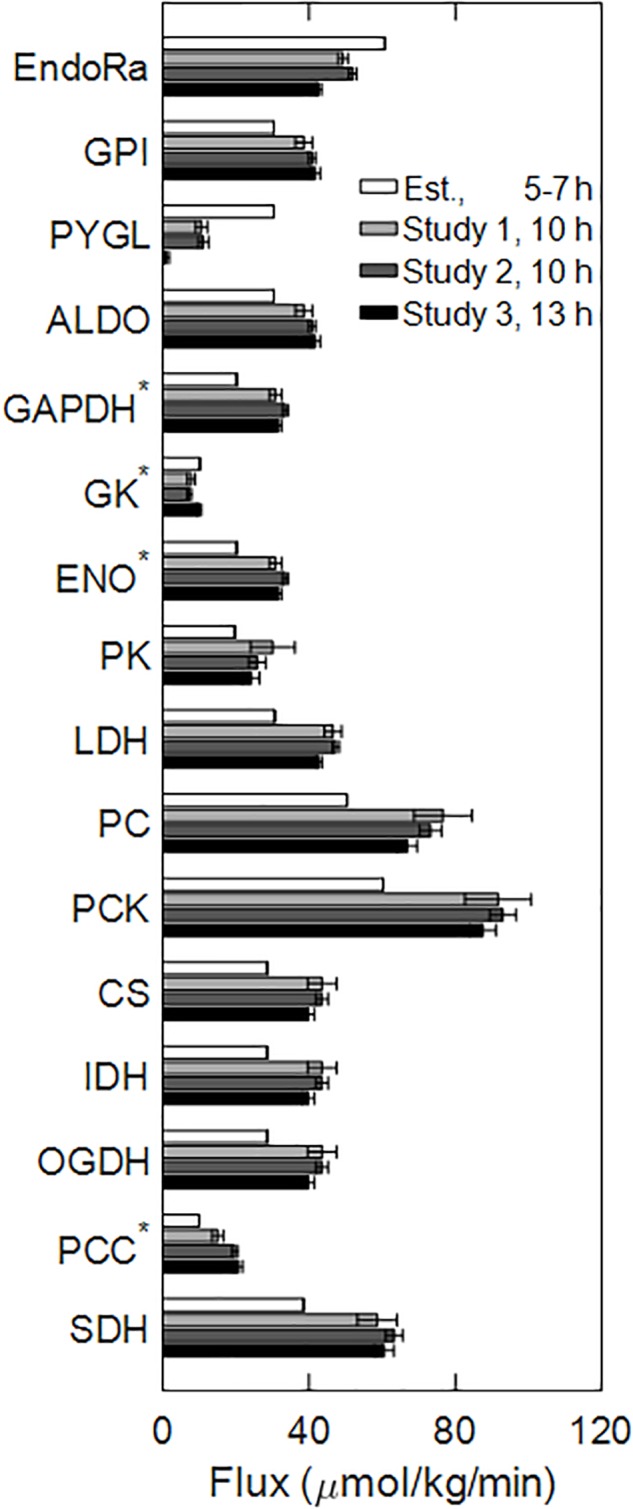
Central carbon metabolic pathway fluxes through the reactions illustrated in Figure 2, measured in Studies 1–3 (10 and 13 h time points), and estimated from the literature at 5–7 h time interval. Bars represent mean flux values, and the error bars represent the SE of the means. The numbers of biological replicates in Studies 1–3 were 9, 8, and 8, respectively. Fluxes labeled by an asterisk (GAPDH, GK, ENO, and PCC) were expressed in hexose units, i.e., divided by a factor of two from their actual values. Abbreviated reaction names on the *y*-axis follow their definitions in the legend for Figure 2.

During fasting, the liver produces glucose by synthesizing it from glycerol, lactate, and amino acids, as well as by breaking down glycogen. Figure 2 shows a schematic of the liver glucose production pathways, which include reactions of glycogenolysis, gluconeogenesis, and the tricarboxylic acid cycle. The aforementioned fluxes are collectively termed central carbon fluxes. The flux values through individual reactions at 10 and 13 h of fasting (Figure 3, Studies 1–3) were measured by stable isotope tracer studies, and those at 5–7 h of fasting (Figure 3, Est. 5–7 h) were compiled from the literature under conditions similar to our studies. In all studies considered for flux values at 5–7 h, food was withdrawn at the beginning of the light cycle. To reduce the influence of potential confounding factors, we first obtained absolute flux of liver glucose production from Rossetti et al.’s (1993) study conducted in 322 g male Sprague-Dawley rats [standard error (*SE*) = 7 g, *n* = 35] fed standard chow under conscious unrestrained conditions. The fractional contribution of glycogen to liver glucose production (48%) at 5–7 h was reported to be invariant to rat strain, body weight, state of anesthesia, and measurement technique (Rossetti et al., 1993; Neese et al., 1995; Peroni et al., 1997; Sena et al., 2007; Jin et al., 2013). The remaining 52% of glucose output came from glycerol, and lactate and amino acids (Rossetti et al., 1993; Neese et al., 1995; Peroni et al., 1997; Sena et al., 2007; Jin et al., 2013). The reported range of glycerol contribution was 15–19% and that of lactate and amino acids was 37–41% (Peroni et al., 1997; Sena et al., 2007; Jin et al., 2013) in various rat strains and a wide range of body weights. We selected fractional contributions of glycerol and lactate from the study of Jin et al. (2013) where they used 324 g male Sprague-Dawley rats (*SE* = 4 g, *n* = 9). Table 2 shows the fractional contributions of various precursors to liver glucose output at 5–7 h and Figure 3 shows the absolute flux values.

**Table 2:** Fractional contributions of metabolic precursors glycogen, glycerol, and lactate and amino acids to liver glucose production at varying durations of fasting.

Metabolic precursor	Percent contribution to liver glucose output		
	
	5–7 h	10 h	13 h
Glycogen	48^a^	20^b^	2.3^b^
Glycerol	15^a^	16^b^	23.4^b^
Lactate and amino acids	37^a^ (33^a^)	64^b^ (51^c^)	74.3^b^ (59^c^)
(lactate)


The fractional contribution of lactate alone to liver glucose production is shown in parentheses adjacent to that of lactate and amino acids in total. ^*a*^(Rossetti et al., 1993; Jin et al., 2013). ^*b*^This study. ^*c*^(Lopez et al., 1998).

Overall glucose output progressively decreased by 30% from 5 to 7 h until 13 h of fasting. Much of this reduction was due to a decrease in the flux of glycogenolysis, whose fractional contribution to glucose output decreased from 48% at 5 h to 2.3% at 13 h of fasting (Table 2). Thus, the contributions of the remaining precursors—glycerol, lactate, and amino acids—to glucose output remained nearly constant as absolute values but increased as fractions of glucose output. As a result, the absolute fluxes through the reactions downstream of glycogen breakdown (PYGL in Figure 2), beginning with glucose-6-phosphate isomerase (GPI in Figure 2) and ending in the tricarboxylic acid cycle at succinate dehydrogenase (SDH in Figure 2), were nearly equal in magnitude at 10 and 13 h of fasting but higher than the values at 5–7 h of fasting (Figure 3).

The major conclusions from the central carbon flux data (Figure 3) were that glycogenolysis and overall glucose output decline with fasting duration. A key observation was that the glycogenolysis flux was almost completely depleted after 13 h of fasting. The flux analysis assumption that liver metabolism operated in a pseudo-steady state at 5–7 h and 10–13 h is consistent with numerous observations reported in the literature (McGarry et al., 1973; Rossetti et al., 1993). The 5–7-h time interval represented the end of an early post-absorptive period—where glycogen breakdown contributed to half of the liver glucose output—which was followed by a steep decline in glycogenolysis and a steep increase in ketogenesis plateauing at the 10–13-h time interval. Although the absolute flux of gluconeogenesis from glycerol was nearly equal at all time points, the flux of gluconeogenesis from lactate and amino acids was higher at the 10–13-h time interval, which indicated the coupling of liver metabolism to extra-hepatic sources of precursors for gluconeogenesis after longer fasting durations. Finally, a key approximation in the central carbon flux analysis was that the liver provided all of the glucose output. Although the kidney is also known to contribute to overall gluconeogenesis, its contribution is important only at fasting durations beyond 24 h (Mithieux et al., 2006). Together with previous evidence, our data suggest the presence of distinct metabolic states after 5–7 h and 10–13 h of fasting.

### Metabolite Changes Observed During Short-Term Fasting

Plasma metabolites changed after short-term fasting (Table 3). Given the similarity in liver central carbon fluxes, we treated the 5-h (Studies 1 and 2) and 7-h (Study 3) fasting durations as early time points, and the 10-h (Studies 1 and 2) and 13-h (Study 3) durations as later time points for determining metabolite fold-change values and their statistical significance. Of the 884 metabolites observed across the three studies, 198 changed significantly (*p* < 0.01). Of these, 39 metabolites were represented in the rat metabolic network model (*iRno*) as exchangeable between liver cells and the extracellular space or plasma. We compared our model predictions for the direction of change with fasting to those for the 39 metabolites, 33 of which showed an increase and 6 of which showed a decrease.

**Table 3:** Observed changes in metabolites between early (5–7 h) and late (10–13 h) time intervals, experimentally measured in the plasma, and in the subset that is represented in the rat metabolic network model as exchangeable between the hepatocyte and plasma.

Metabolite set	Total Elevated	Depressed (*p* < 0.01)	(*p* < 0.01)
Experimentally measured in plasma	824	121	72
Model represented and exchangeable	216	33	6
with plasma


We also compared the significant changes in plasma metabolites observed in the present study to those reported in the literature on short-term fasting in the rat (McGarry et al., 1973; Ho, 1976; Brass and Hoppel, 1978; Palou et al., 1981; Kotal et al., 1996; Ikeda et al., 2014). In terms of major metabolite pathways, most of the changes reported in the literature were in agreement with those found in our study (Table 4). Important changes indicative of fasting were a reduction in glucose and phospholipids, and an elevation of ketone bodies, fatty acyl carnitines, corticosterone, and choline. Furthermore, key liver-specific metabolite changes observed here and in the literature were the elevation of primary and secondary bile acids, and the elevation of bile pigments bilirubin and biliverdin. Supplementary Table S3 provides detailed lists of those metabolites and the entire summary of statistical analysis of all metabolites.

**Table 4:** Concordance of observed changes in plasma metabolite data with reported changes in the literature due to short-term fasting.

Pathway	Number of metabolites reported in this study (and in the literature)			Fraction in agreement with this study	Reference
			
	Elevated	Unchanged	Depressed		
Amino acid	3 (6)	18 (13)	1 (3)	0.6	Palou et al., 1981
Carbohydrate	0	0	1 (1)	1.0	McGarry et al., 1973; Palou et al., 1981
Hemoglobin and porphyrin	3 (3)	0	0	1.0	Kotal et al., 1996
metabolism
Lipid/carnitine	0 (0)	0	1 (0)	1.0	Brass and Hoppel, 1978
Lipid/corticosteroids	1 (1)	0	0 (0)	1.0	Dauchy et al., 2010; Ikeda et al., 2014
Lipid/diacylglycerol	1 (0)	0	7 (8)	0.9	Ikeda et al., 2014
Lipid/acyl carnitine	23 (23)	0	0	1.0	Brass and Hoppel, 1978
Lipid/ketone bodies	2 (2)	0	0	1.0	McGarry et al., 1973; Palou et al., 1981
Lipid/phosphatidylcholine	0	0	11 (11)	1.0	Ikeda et al., 2014
Lipid/phosphatidylinositol	0	0	5 (5)	1.0	Ikeda et al., 2014
Lipid/choline	1 (1)	0	0	1.0	Ikeda et al., 2014
Lipid/bile acids	9 (9)	0	0	1.0	Ho, 1976
Lipid/sterol	0	0	1 (1)	1.0	Ikeda et al., 2014


Reports on large-scale data on plasma metabolite changes during a short-term fast, the number of biological replicates required to resolve them, and their sensitivity to the type of vehicle administered, do not exist in the literature. The number of metabolites measured in Studies 1, 2, and 3 were 569, 645, and 633, respectively, where the vehicle administered to the rats was different for each study. The metabolite fold-change values needed to be pooled across the three studies to resolve metabolite changes above experimental and biological noise during short-term fasting. The sum total of unique metabolites measured in the plasma in all three studies was 824 (Table 3), of which 420 were common to all three studies, 183 were common to exactly any two studies, and 221 were observed in exactly any one study. We calculated bootstrapped 99% confidence intervals of the fold-change values of the 420 common metabolites and confirmed that the vehicle was not a significant factor influencing metabolite changes (see Supplementary Table S3).

Among the 193 significantly changed metabolites (Table 3), 104 (54%) were measured in all three studies, 44 (23%) in exactly any two studies, and 45 (23%) in exactly any one study. Similarly, among the 631 unchanged metabolites, 316 (50%) were measured in all three studies, 139 (22%) in exactly any two studies, and 176 (27%) in exactly any one study. Taken together, there was no study-wise representation bias in the proportion of metabolites among the changed and unchanged groups, nor was there any differential effect of the vehicle on metabolite changes between studies, ensuring that pooling of metabolite fold-change data across studies was not confounded by known experimental differences between studies.

Of the 216 metabolites represented in *iRno* as exchangeable metabolites, 163 (76%) were measured in all three studies, which indicated the overall reliability of the data on exchangeable metabolites. Similarly, among the 39 significantly changed metabolites, 35 (90%) were measured in all three studies, which indicated the reliability of the metabolite data against which our model predictions were compared. Of the remaining four, *N*-carbamoylaspartate was measured in Study 3, acetylcarnitine in Study 1, inosine in Studies 1 and 2, and isocitrate in Studies 1 and 3.

Metabolite pathway annotations showed that lipids, amino acids, and cofactors and vitamins account for 49%, 23%, and 4% of the 824 metabolites, respectively, which indicated that lipid metabolites constituted the single largest category. Among the 193 metabolites that changed significantly, lipid metabolites again constituted the single largest group at 58%. The fraction of significantly changed lipid metabolites among all lipid metabolites was also highest at 28%, when compared to changes in other major pathways (19% or less). These results underscore the significance of lipids during short-term fasting.

### Metabolic Gene Expression Did Not Change Significantly During Short-Term Fasting

Gene expression changes in the liver during short-term fasting in all three studies (Table 5) revealed that the transcripts from each study mapped to a similar total number of genes (about 14,000), of which 2,258 were mapped to 2,240 in *iRno*. Out of the 2,325 genes in *iRno*, which were annotated with NCBI gene identifiers, 2,240 had 2,258 ENSEMBL gene identifiers that were used to annotate our transcriptomic data, with several genes mapping to than one ENSEMBL identifier. Based on the criteria of a false discovery rate of less than 0.1 and a biological effect size cutoff of 0.6 (corresponding to the 90th percentile), we found no statistically and biologically significant change in the expression of metabolic genes mapping to *iRno* in Studies 1 and 3 except for 100 genes in Study 2. Therefore, we did not use any differential gene expression-based weights in our implementation of the TIMBR algorithm to predict plasma metabolite changes. Supplementary Table S4 shows the results of the gene expression analysis.

**Table 5:** Summary of gene expression changes with fasting.

Study	Number of genes		
	
	Total	Mapped to *iRno* (total)	Mapped to *iRno* (*q* < 0.1)
1	14,115	2,240	0
2	14,581	2,240	100
3	14,419	2,240	0


### Liver Metabolism Accounts for 64% of Plasma Metabolite Changes

**FIGURE 4:**
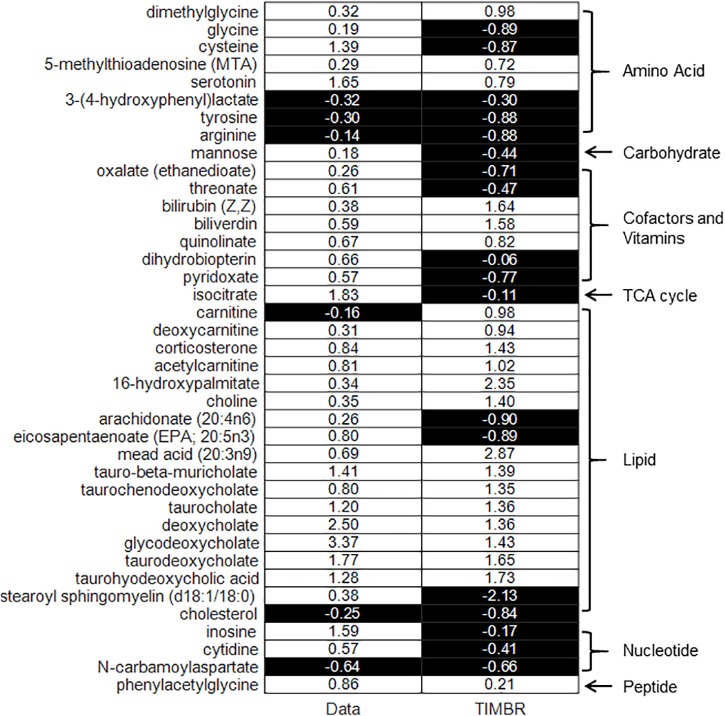
Binary heat map of TIMBR scores of significantly changed exchangeable metabolites in plasma represented in *iRno* compared with measured fold-change values, grouped by major biochemical pathways: amino acid, carbohydrate, cofactors, and vitamins, TCA cycle, lipid, nucleotide, and peptide. The values in the left-hand side column (data) are measured log_2_(fold change) values of metabolites, grouped as depressed (black background), or elevated (white background) metabolites. The values in the right-hand side column are the computed TIMBR scores whose negative (black background) or positive (white background) sign indicates a predicted tendency of the metabolite to be depressed or elevated in plasma.

We integrated liver central carbon flux data, as well as known physiological flux bounds for metabolite exchange fluxes at early (after 5–7 h of fasting) and late (after 10–13 h of fasting) time points, with *iRno* using the TIMBR algorithm. We then used the TIMBR algorithm to compute a TIMBR score, whose positive or negative sign indicated the tendency of a metabolite to increase or decrease in the plasma, respectively, owing to changes in the liver metabolic network demand induced by fasting. The TIMBR predictions agreed overall with the metabolite changes observed here; TIMBR scores accurately predicted five out of six depressed, and 20 out of 33 elevated metabolites (Table 6). A summary of the 39 metabolites, their observed log_2_(fold-change) values, and corresponding TIMBR scores (Figure 4) revealed an overall accuracy of 64% for predicting any changes, and accuracies of 61% and 83% for predicting elevated and depressed metabolites, respectively. The probability that 64% or higher prediction accuracy could be achieved by chance was calculated to be 0.054, using the exact binomial test. Therefore, our network model of liver metabolism could account for 64% of plasma metabolite changes (increase or decrease) that were represented in the model, during short-term fasting.

**Table 6:** Concordance of TIMBR predictions with observed directions of change in metabolite data.

Direction of change	Number of metabolites measured	Concordant model predictions
Elevated	33	20
Depressed	6	5
All	39	25


The results in Figure 4, organized by metabolite pathways, revealed three major pathways represented in our data set: amino acids (8 metabolites), cofactors and vitamins (7 metabolites), and lipids (18 metabolites). The model accuracy in predicting metabolite changes for these three major pathways was 75% for amino acids, 42% for cofactors and vitamins, and 78% for lipids, providing estimates of both the reliability of the network model and the hepatic origin of metabolite changes in the pathways. In particular, the model achieved 100% accuracy in predicting the elevation of five primary and secondary bile acids (under lipids in Figure 4), and two bile pigments (under cofactors and vitamins), which are specific to the liver.

### Computational Model Assumptions, Limitations, and Interpretation of Predictions

The rat metabolic network model, *iRno*, currently the most comprehensive genome-scale model of rat metabolism, instantiated with physiological flux bounds pertinent to the liver, was tested for satisfying defined liver-specific metabolic functionalities (Blais et al., 2017). The implicit assumption in our model was that overall liver metabolism could be represented by a single network with a representative set of physiological boundary conditions. This assumption seemed to contradict the known metabolic differences in hepatocytes between perivenous and periportal regions in the liver (Thurman et al., 1986). Despite not representing those different kinds of hepatocytes in our model, the overall satisfaction of liver metabolic tasks attested to a sufficient representation of liver metabolic functions originating in both regions. Additionally, the physiological flux bounds and central carbon fluxes employed to constrain the model did not include any metabolic heterogeneity. Finally, a key assumption in analyzing the model was that the network maintained a steady state, which was reasonable given the known metabolic flux conditions at 5–7 h and 10–13 h.

A limitation of our modeling analysis was the restricted coverage of metabolites exchanged between the plasma and liver cells. Additional curation of *iRno*, which included addition of exchange fluxes to improve network coverage of plasma metabolites, was limited by the paucity of literature evidence on the exchangeability of those metabolites. Consequently, the fraction of lipid metabolites among the 216 exchangeable metabolites (37%) was lower than that of the overall data set (49%). However, the fraction of lipid metabolites among the 39 significantly changing metabolites was higher at 46%, which is consistent with the trend in lipid metabolite fractions observed in the overall data set. Therefore, metabolite changes mapped to the network model are not biased by their limited coverage.

The measured changes in the circulating metabolites in plasma reflected the fasting response of the whole body. Our modeling effort sought to investigate plasma metabolite changes that can be associated with changes in liver metabolism under short-term fasting conditions where the primary observation was a decrease in the hormonally regulated flux of liver glycogenolysis and no significant transcriptomic changes of liver enzymes (Lin and Accili, 2011). Our metabolic network analysis was made liver specific and relevant to liver metabolism by the flux constraints. We used the *in vivo* central carbon fluxes derived from our tracer-infusion studies under short-term fasting conditions coupled with literature data from several studies during short-term fasting that sets the overall metabolite uptake and secretion fluxes of the liver (Lopez et al., 1998; Jin et al., 2013). This analysis assumed that the bulk of the glucose production flux captured by the *in vivo* metabolic flux analysis was of hepatic origin under these conditions (Hasenour et al., 2015). Thus, even though the measured metabolite changes were reflective of the overall systemic response, our computational analysis estimated those changes that were in concordance with a hepatic origin. To assess the impact of liver transcriptomic changes, we repeated our implementation of the TIMBR method using all of the transcriptomic changes regardless of their statistical significance and found that the predicted directions of metabolite changes were unaltered from those shown in Figure 4 (see Supplementary Figures S1–S3).

Finally, the estimated model accuracy in predicting bile acids and bile pigments (100%, *p* = 0.004, subset of lipids), lipids (78%, *p* = 0.03), and amino acids (75%, *p* = 0.29) demonstrated the capability of the model to describe liver metabolic functions, and provided estimates of contributions of liver metabolism that agreed with metabolite changes observed in those pathways. In particular, lipid metabolite changes emerged as indicators of changes in liver metabolism, which were characterized both experimentally and computationally with sufficient statistical significance.

## Conclusion

Liver glycogenolysis became vanishingly small over the course of a short-term fast of 13 h, which resulted in a decline in the overall liver glucose output from 5 h until 13 h. Metabolites in plasma during this period showed changes known to be associated with short-term fasting, whereas liver gene expression did not change significantly. Finally, our computational analysis showed that two-thirds of the metabolite changes in plasma between 5–7 h and 10–13 h of fasting could be explained by central carbon flux changes in the liver without significant changes in gene expression.

## Data Availability

RNA-seq datasets generated for this study can be found in the Gene Expression Omnibus repository (accession numbers GSE123935, GSE124004, and GSE123987). Metabolomics data sets generated for this study are provided in Supplementary Table S3.

## Author Contributions

KV conceived the short-term fasting analysis, analyzed RNA-seq and metabolomics data, computed model predictions, and wrote the manuscript. VP curated the rat metabolic network model. MW and MR performed metabolic flux calculations based on isotope labeling measurements. SE performed all of the animal studies, including catheterization surgeries. IT processed plasma samples for metabolic flux analysis. TO collected and analyzed all of the blood samples. RP contributed to RNA extraction from tissue and purifications. JR helped to conceive and supervise the study, and helped to edit the manuscript. MS conceived the study, supervised and carried out the experiments on rats to generate the raw data, and helped to write the manuscript. JY conceived the study, supervised the metabolic flux analysis, and helped to write the manuscript. AW conceived and supervised the study, and helped to edit and write the final manuscript.

## Conflict of Interest Statement

The authors declare that the research was conducted in the absence of any commercial or financial relationships that could be construed as a potential conflict of interest.

## Supplementary Material

FIGURE S1 Binary heat map of TIMBR scores of significantly changed exchangeable metabolites in plasma represented in *iRno* compared with measured fold-change values, computed using both liver physiological flux changes and gene expression changes in Study 1 and grouped by major biochemical pathways: amino acid, carbohydrate, cofactors, and vitamins, TCA cycle, lipid, nucleotide, and peptide. The values in the left-hand side column (data) are measured log_2_ (fold change) values of metabolites, grouped as depressed (black background), or elevated (white background) metabolites. The values in the right-hand side column are the computed TIMBR scores whose negative (black background) or positive (white background) sign indicates a predicted tendency of the metabolite to be depressed or elevated in plasma.

FIGURE S2 Binary heat map of TIMBR scores of significantly changed exchangeable metabolites in plasma represented in *iRno* compared with measured fold-change values, computed using both liver physiological flux changes and gene expression changes in Study 2 and grouped by major biochemical pathways: amino acid, carbohydrate, cofactors, and vitamins, TCA cycle, lipid, nucleotide, and peptide. The values in the left-hand side column (data) are measured log_2_ (fold change) values of metabolites, grouped as depressed (black background), or elevated (white background) metabolites. The values in the right-hand side column are the computed TIMBR scores whose negative (black background) or positive (white background) sign indicates a predicted tendency of the metabolite to be depressed or elevated in plasma.

FIGURE S3 Binary heat map of TIMBR scores of significantly changed exchangeable metabolites in plasma represented in *iRno* compared with measured fold-change values, computed using both liver physiological flux changes and gene expression changes in Study 3 and grouped by major biochemical pathways: amino acid, carbohydrate, cofactors, and vitamins, TCA cycle, lipid, nucleotide, and peptide. The values in the left-hand side column (data) are measured log_2_ (fold change) values of metabolites, grouped as depressed (black background), or elevated (white background) metabolites. The values in the right-hand side column are the computed TIMBR scores whose negative (black background) or positive (white background) sign indicates a predicted tendency of the metabolite to be depressed or elevated in plasma.

TABLE S1 Rat genome-scale metabolic network reconstruction *iRno*, with modifications.

TABLE S2 Flux constraints for *iRno* during short-term fasting obtained from the literature.

TABLE S3 Metabolomic imputed data from Studies 1–3, and the summary of statistical analysis of metabolite changes during short-term fasting in all three studies.

TABLE S4 Results of RNA-seq data analysis, using the Wald and the likelihood ratio tests, to assess gene expression changes during short-term fasting in Studies 1–3.

DATA SHEET S1 Rat genome-scale metabolic network reconstruction *iRno* in SBML format.

DATA SHEET S2 Computer code to analyze metabolomics data.

DATA SHEET S3 Computer code to reproduce TIMBR analysis results in Figure 1 and Supplementary Figures S1–S3.
