# Supplementary figures and images for: Network Modeling of Liver Metabolism to Predict Plasma Metabolite Changes During Short-Term Fasting in the Laboratory Rat
Source: Front Physiol. 2019 Mar 1;10:161. doi: 10.3389/fphys.2019.00161 (PMC6405515; doi:10.3389/fphys.2019.00161)

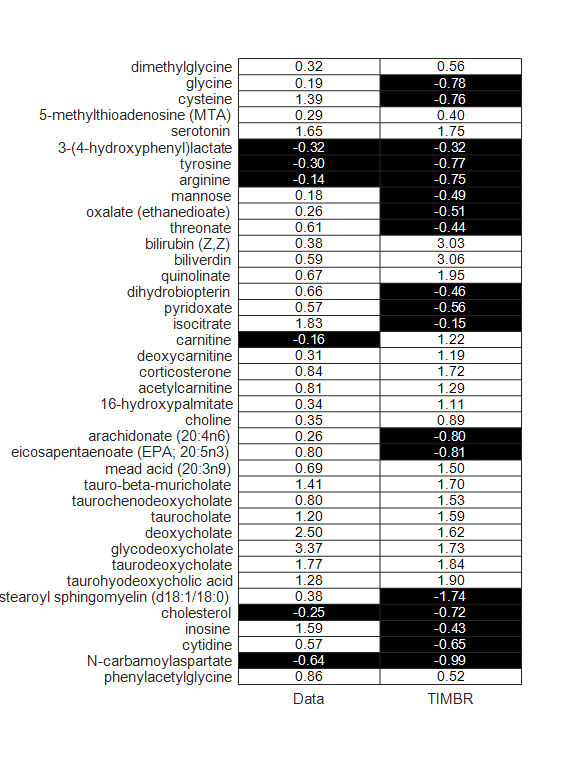

Supplement: FIGURE S1 — Binary heat map of TIMBR scores of significantly changed exchangeable metabolites in plasma represented in iRno compared with measured fold-change values, computed using both liver physiological flux changes and gene expression changes in Study 1 and grouped by major biochemical pathways: amino acid, carbohydrate, cofactors, and vitamins, TCA cycle, lipid, nucleotide, and peptide. The values in the left-hand side column (data) are measured log2 (fold change) values of metabolites, grouped as depressed (black background), or elevated (white background) metabolites. The values in the right-hand side column are the computed TIMBR scores whose negative (black background) or positive (white background) sign indicates a predicted tendency of the metabolite to be depressed or elevated in plasma. [file Image_1.tif]

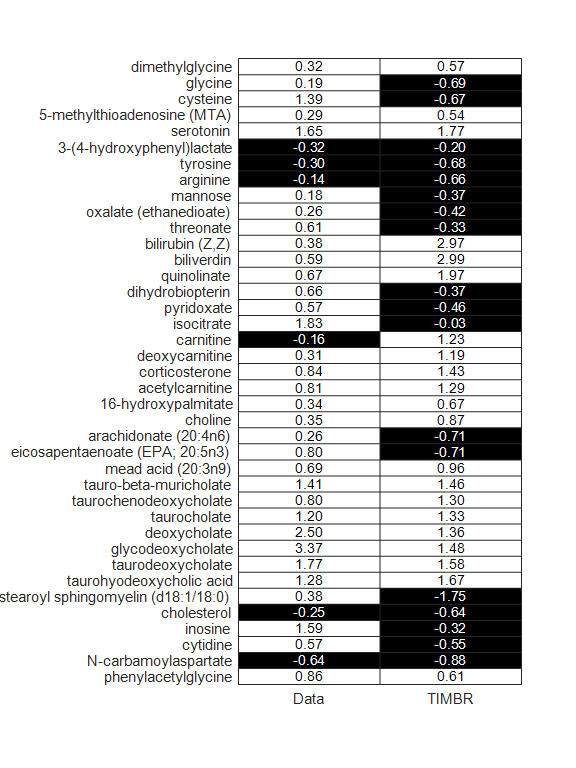

Supplement: FIGURE S2 — Binary heat map of TIMBR scores of significantly changed exchangeable metabolites in plasma represented in iRno compared with measured fold-change values, computed using both liver physiological flux changes and gene expression changes in Study 2 and grouped by major biochemical pathways: amino acid, carbohydrate, cofactors, and vitamins, TCA cycle, lipid, nucleotide, and peptide. The values in the left-hand side column (data) are measured log2 (fold change) values of metabolites, grouped as depressed (black background), or elevated (white background) metabolites. The values in the right-hand side column are the computed TIMBR scores whose negative (black background) or positive (white background) sign indicates a predicted tendency of the metabolite to be depressed or elevated in plasma. [file Image_2.tif]

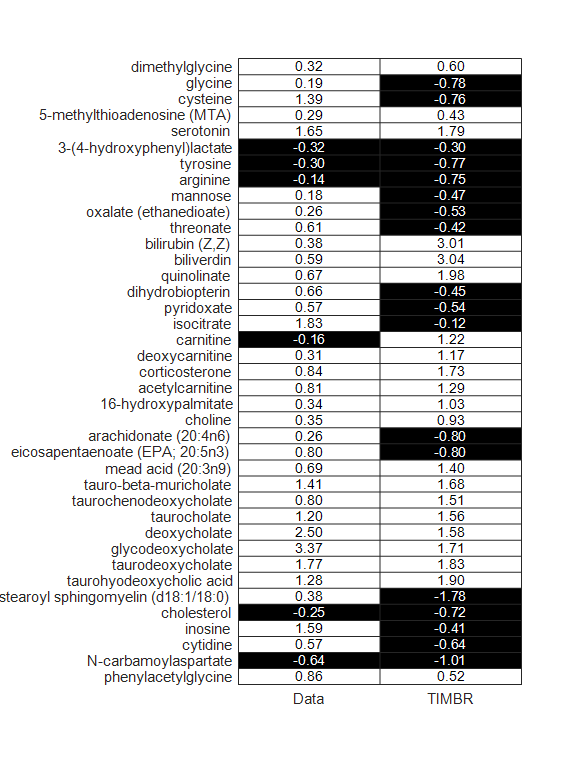

Supplement: FIGURE S3 — Binary heat map of TIMBR scores of significantly changed exchangeable metabolites in plasma represented in iRno compared with measured fold-change values, computed using both liver physiological flux changes and gene expression changes in Study 3 and grouped by major biochemical pathways: amino acid, carbohydrate, cofactors, and vitamins, TCA cycle, lipid, nucleotide, and peptide. The values in the left-hand side column (data) are measured log2 (fold change) values of metabolites, grouped as depressed (black background), or elevated (white background) metabolites. The values in the right-hand side column are the computed TIMBR scores whose negative (black background) or positive (white background) sign indicates a predicted tendency of the metabolite to be depressed or elevated in plasma. [file Image_3.tif]
